# Histologic Analysis of ‘Distraction Vaginogenesis’ in a Rat Model

**DOI:** 10.3390/pathophysiology31020022

**Published:** 2024-06-08

**Authors:** Hannah Meyer, Lexus Trosclair, Sean D. Clayton, Collyn O’Quin, Carol Crochet, Joshua C. Colvin, Valerie Welch, Ahmed Alhaque, Giovanni Solitro, Mila Shah-Bruce, J. Steven Alexander, Donald L. Sorrells

**Affiliations:** 1 Department of Surgery, LSU Health Shreveport, Shreveport, LA 71103, USA; 2 Department of Pathology, LSU Health Shreveport, Shreveport, LA 71103, USA; 3 Department of Molecular and Cellular Physiology, LSU Health Shreveport, Shreveport, LA 71103, USAjonathan.alexander@lsuhs.edu (J.S.A.); 4 Department of Orthopedic Surgery, LSU Health Shreveport, Shreveport, LA 71103, USA; 5 Department of Obstetrics and Gynecology, LSU Health Shreveport, Shreveport, LA 71103, USA

**Keywords:** vaginal agenesis, vaginal atresia, vaginal dilation, vaginal elongation, Mayer-Rokitansky-Küster-Hauser syndrome, tissue reconstruction, vaginal histology

## Abstract

Vaginal agenesis (VA) is frequently associated with mullerian agenesis. VA treatments include mechanical dilation and surgical vaginoplasty. We created a vaginal expansion sleeve (VES) as a novel device to progressively lengthen the vaginal canal. This study evaluated the histologic effects of the VES on rat vaginal tissue. The VES is a spring-like device made of proprietary woven cylindrical material and flat resin caps. The VESs were constructed as 25–30 mm, pre-contracted springs, which were secured into the vaginas of six Sprague Dawley rats and allowed to re-expand post-surgically. After one week, the VESs were removed, and the vaginas were harvested and measured in length. Test (*n* = 6) and control (*n* = 4) formalin-fixed paraffin-embedded tissues were stained with hematoxylin and eosin (H&E), Masson’s trichrome, and anti-Desmin antibodies. The VESs achieved significant vaginal lengthening. The mean vaginal canal length increased from 20.0 ± 2.4 mm to 23.8 ± 1.2 mm after removal of the VESs (*n* = 6, *p* < 0.001), a 19% increase. There was a positive correlation between the expander/tension generated in the vagina and the amount of acute and chronic inflammation. H&E staining revealed increased submucosal eosinophilia in five of the six test tissues. One VES sample that was lengthened to 30 mm long showed evidence of lymphocytic and neutrophilic inflammation. Desmin immunostaining and Masson’s trichrome stain revealed a thinner muscularis with more infiltrative fibrous tissue between muscle fibers in the test tissue compared to the control tissue. Although effective, the VES may provoke at least a transient increase in eosinophils consistent with a localized immune reaction during muscularis remodeling.

## 1. Introduction

Vaginal atresia refers to the congenital absence or shortening of the vagina and is commonly observed in genetic conditions such as Mayer-Rokitansky-Küster Hauser (MRKH) syndrome and complete androgen insensitivity syndrome (CAIS) [1,2]. MRKH syndrome occurs due to incomplete embryonic development of the mullerian ducts and mullerian tubercle, leading to uterine and/or vaginal agenesis or atresia [3,4]. Infants with MRKH syndrome, who have a chromosomal makeup of 46XX, typically have externally normal-looking genitalia and develop secondary sex characteristics normally as a result of functioning ovaries [5,6]. MRKH syndrome is primarily classified into two types. Type I MRKH syndrome involves no associated extragenital malformations, whereas type II does, commonly associated with renal and skeletal system pathologies [6]. Androgen insensitivity (AIS) is a rare X-linked recessive disorder of the androgen receptors with a 46XY karyoptye [7]. In CAIS, patients present either in infancy as an inguinal swelling containing a testis or in adolescence as primary amenorrhea [8]. Internally, the uterus, cervix, and proximal vagina are absent due to the action of the anti-Mullerian hormone produced by Sertoli cells in the testis [8]. If left untreated, vaginal atresia can significantly impact emotional well-being and quality of life due to feelings of being ’different’ or ’unusual’. Therefore, treatment is imperative when the patient is emotionally prepared [5,9]. The current treatment options for vaginal atresia include surgical vaginoplasty and non-surgical primary dilation therapy. 

Surgical vaginoplasty encompasses several procedural techniques, including the Abbe–McIndoe, Davydov, intestinal vaginoplasty, and Vecchietti procedures [10,11]. The Abbe–McIndoe method involves dissecting out the neovaginal space between the bladder and the rectum and then covering it with a split-thickness skin graft [10,11,12,13]. Complications and side effects associated with this technique include graft rejection, high rates of neovaginal stenosis, and lack of lubrication [10,11]. The Davydov technique is a modification of the Abbe–McIndoe procedure where the peritoneum is used as the neovaginal vault lining and thus does not involve a scar from the skin graft site [10,11,14,15]. As such, complications associated with this technique are similar to that of the Abbe–McIndoe. Intestinal vaginoplasty involves using a segment of the bowel (typically the sigmoid colon, ileum, or jejunum) to create the neovaginal canal [10,11]. This method is unique because it does not require post-operative dilation therapy or synthetic lubrication [11]. However, complications associated with this technique include prolapse, excessive discharge, bowel obstruction, and post-operative ileus [10,11]. Finally, the less invasive Vecchietti method involves the progressive upward traction of an acrylic 2 cm olive-shaped bead that is placed on the vaginal dimple [10,11,16,17]. Laparoscopically placed threads run cephalad preperitoneally along the abdominal wall and are connected to a traction device placed on the patient’s external abdomen [10,11]. These threads are tightened daily, with a traction period of one week [10,11,16,17]. Complications associated with this technique include lesions to the bladder or rectum by the traction threads as well as vaginal prolapse [10,11]. The biggest downside to this method is the pain associated with traction, which requires the patient to remain in the hospital throughout the entire traction process [10,11]. Surgical treatment methods generally carry a higher risk of serious complications compared to traditional dilation therapy, including stenosis, strictures, fistulas, hemorrhage, and prolapse [10,11,18]. 

Due to the degree of invasiveness, these types of surgical vaginoplasties mean that many patients require a prolonged hospital stay. Additionally, regular post-operative dilation therapy after surgical vaginoplasty is typically required to maintain the patency of the newly created vaginal canal, except in the case of intestinal vaginoplasty [11]. Due to the elevated risk of complications and the physical stress associated with surgery, it is considered a secondary treatment option when primary dilation therapy fails to achieve successful neovagina formation.

Mechanical dilation therapy (MDT) is currently recognized as the preferred treatment option for vaginal atresia due to its cost effectiveness and safety compared to surgical intervention [3]. Frank first described MDT in 1938, which involves the use of progressively increasing length and width vaginal molds to create a neovagina [19]. MDT requires the application of pressure using these rigid vaginal molds in the vaginal canal for approximately thirty minutes three times per day, with an average length of treatment of five to six months in highly compliant patients [5,20,21]. After the initial education on proper technique, patients are typically responsible for conducting self-treatment at home, making it exceedingly dependent on patient execution.

We consider treatment success to be both anatomical and functional. Anatomical success, which is generally defined as a vaginal canal with adequate and appropriate length, measures approximately 7 cm in length post-treatment [22]. Functional success is defined by the ability to achieve satisfactory penetrative intercourse or by the ability to receive the largest vaginal dilator without discomfort or pain and is considered to be more meaningful than anatomical success [22,23]. Although many patients have success with first-line MDT, surgical vaginoplasty has been found to have a higher rate of functional success over MDT [24]. Many patients with MRKH syndrome have only a vaginal dimple at the start of treatment, but studies have shown that there is no correlation between initial vaginal length and success with MDT [23,25]. Rather, success in creating adequate vaginal length depends on the frequency of dilation treatments and a patient’s motivation and compliance with treatment daily [2]. One study found that a greater increase in vaginal length, a higher rate of functional success, and a shorter duration of treatment were all achieved in patients that consistently conducted MDT [22]. Therefore, it is crucial to offer psychological assessment and counseling to patients before and throughout the treatment process [2,20]. One of the significant challenges associated with dilation therapy is the constant reminder of the abnormality, which can negatively impact patient motivation and result in unsuccessful neovagina creation [4]. Removing this emotional barrier may increase the likelihood of successful neovagina creation without the need for surgery.

We originally described the approach of ‘distraction vaginogenesis,’ which was initially based on our report of ‘distraction enterogenesis,’ in a preliminary study using ex vivo tissue that investigated tissue remodeling produced by deliberate canal elongation [26,27]. This approach relies on applying distractive mechanical forces using longitudinal expansion devices to lengthen the vaginal canal without causing adverse effects on the tissue [26,27,28].

In this study, we assessed the method of ‘distraction vaginogenesis’ for tissue elongation by utilizing a vaginal expansion sleeve (VES). We aim for our device to be the bridge between at-home dilation therapy and in-hospital surgical treatment. The primary objectives of this study were to evaluate the effectiveness of the VES device and to histologically examine the lengthening of the vaginal wall and assessed tissue events that may occur due to prolonged implantation of the device. Elongation of the vaginal canal with the VES technique may represent an important novel method for producing tissue elongation in MRKH syndrome and other forms of gynecological atresia in which canal elongation is necessary.

## 2. Materials and Methods

### 2.1. VES Design

**Figure 1:**
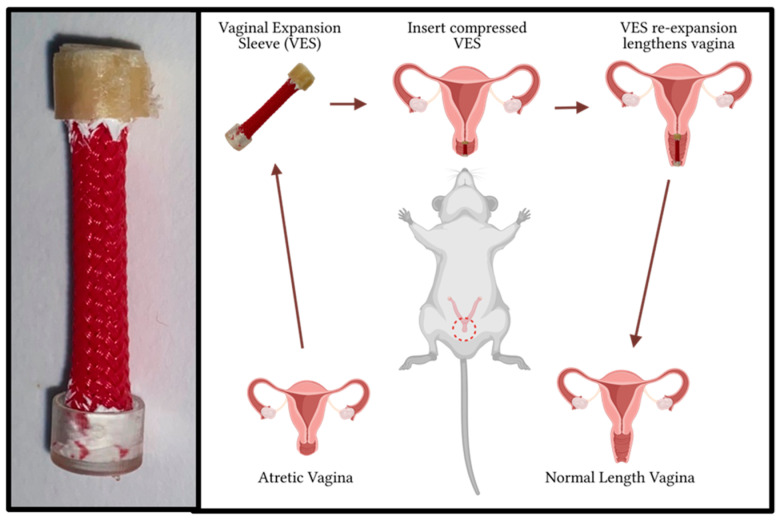
The VES device and its basic implementation in the treatment of vaginal atresia.

This study was approved by the Institutional Animal Care and Use Committee (IACUC) of the Louisiana State University School of Medicine in Shreveport (protocol code P-23-027; approved on 1 February 2023). Our vaginal expansion sleeve (VES) is made of a woven 5 mm cylindrical layered polyethylene terephthalate with helicoid trusses characterized by isometric ends (Figure 1). Two VES devices were cut to 25 mm in length, three to 27 mm in length, and one to 30 mm in length. The variation in VES lengths was to accommodate for the variation in starting length of the individual vaginal canals. Five VES devices had a flat non-absorbable cap comprising the Biocompatible Photopolymer Resin Surgical Guide (Formlabs, Somerville, MA, USA) on the distal end and a flat absorbable cap composed of PVA filament (Prusa Research, Prague, Czech Republic) on the proximal end. The absorbable cap was added to facilitate a smoother insertion without fraying of the sleeve; once dissolved, the sleeve is more easily allowed to expand. To further prevent fraying of the woven material, the ends of the sleeve were coated with liquid rubber prior to attachment of the caps.

### 2.2. Perioperative and Vaginal Lengthening Periods

**Figure 2:**
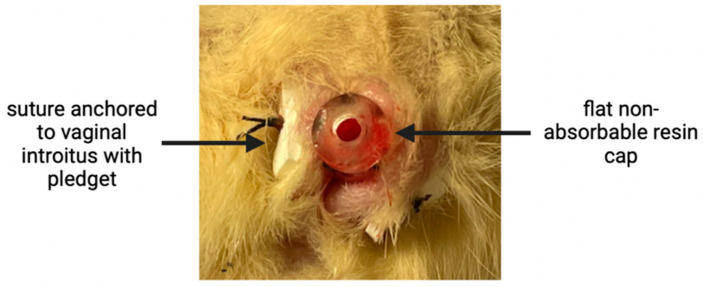
VES inserted and anchored in the rat vaginal canal.

Each rat was anesthetized with isoflurane prior to and during the procedure. A toe pinch test was used to determine proper anesthetization before beginning. Initial vaginal canal lengths were measured using a pediatric anal dilator (Specialty Surgical Products, Victor, MT, USA). A previous study determined that contracting the device to 50% of its original length provided the highest re-expansive force load [26]. The device was first manually pre-contracted and secured into the optimal 50% compression position by looping two sutures through the patent center of the sleeve and tying them tightly on both sides longitudinally. The sleeve was then inserted into the vaginal canal and anchored into place using three non-absorbable 3–0 silk sutures as well as surgical pledgets on the exterior end of the vaginal canal (Figure 2). The compressing ties were then cut to allow for device expansion. For pain relief, a 2.5 mg carprofen/mL saline solution was prepared, and 5 mg/kg body weight was administered subcutaneously at the end of surgery and provided as needed if the rat was in clear distress. Each rat’s torso was wrapped with Coban and surgical tape to limit flexibility and inhibit the rat from reaching the sutures to displace the VES. Loosening wraps were reinstalled halfway through the trial (day 3) to re-establish rigidity. Although some mobility had to be limited, the rats’ ability to access food and water and move around their cages was monitored and maintained throughout the trial. The experimental period was set to seven days, as one rat week is equal to six human months—the average length of treatment for mechanical dilation therapy [29]. On day 7, each rat was euthanized using carbon dioxide followed by thoracotomy, after which the devices were removed. Post-intervention vaginal canal lengths were measured using the same pediatric anal dilator used to take the initial measurements. A statistical analysis of the vaginal canal measurements was accomplished using Microsoft Excel Version 16.77.1.

### 2.3. Histological Analysis

Vaginal canals were then harvested from all six test rats and four control rats, and tissue samples were prepared for histologic analysis. Vaginal canals were transected, rolled from the cervical end to the introitus around a wooden stick, and pinned before sectioning—the so-called ‘Swiss roll’ technique [30]. Tissue sections were fixed in 3.7% phosphate buffered formaldehyde for 24 h before being transferred to the anatomic pathology lab. Following overnight fixation and processing, the tissues were embedded in paraffin and affixed to glass slides. Routine hematoxylin and eosin staining was performed. Trichrome stains and Desmin immunohistochemical stains were also performed on the tissue samples. Slides were reviewed and photographs recorded by a board-certified anatomic pathologist.

## 3. Results

### 3.1. Vaginal Lengthening with VES

Six VES devices were inserted into the vaginal canals of live Sprague Dawley rats and allowed to expand tissue for 7 days. Four VES devices had extruded from the vaginas on day 6. Two devices remained in place until the end of the trial at day 7. The mean initial vaginal canal length was 20.00 ± 2.45 mm. At the end of the trial, the mean expanded vaginal canal length was 23.83 ± 1.17 mm (*p* < 0.001), resulting in a mean 20.41 ± 0.14% increase (Table 1).

The weight of each rat was recorded at the beginning and end of the trial. No significant changes in weight were observed, but this effort was made due to the rat’s decreased mobility and our increased attention to their food access.

### 3.2. Histologic Analysis

**Figure 3:**
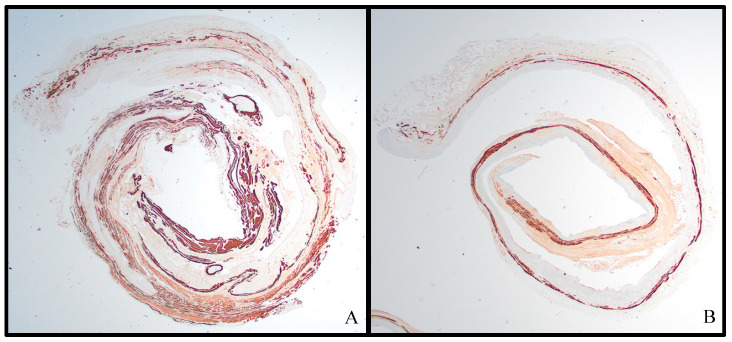
Desmin-immunostained Swiss rolls at 20× magnification demonstrating thinned muscularis layer: (**A**) control sample 2; (**B**) VES test sample.

**Figure 4:**
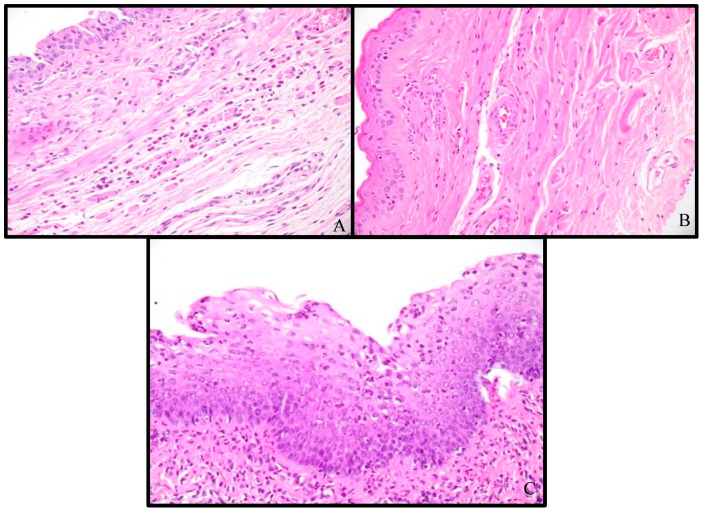
H&E stain at 400× magnification demonstrating inflammatory infiltrate in (**A**) control sample 4, (**B**) test sample 4, and (**C**) test sample 6.

H&E slides from the four control samples and six test samples were reviewed and noted to have differences in vaginal wall thickness and inflammatory infiltrate. The vaginal tissues from the VES experimental rats showed an overall decrease in vaginal wall thickness (Figure 3), with a mean control tissue thickness of 0.54 mm (SD = 0.15 mm) and a mean test tissue thickness of 0.387 mm (SD = 0.084 mm), a 28% thinning over the course of 1 week (*p* = 0.07, paired Student *t*-test). All but one test sample was also shown to have an increase in inflammatory infiltrate, primarily submucosal eosinophilic inflammation (Figure 4). Eosinophils were quantified in the densest high power field (HPF, 400×) in the control and test tissues. In the control tissues, there was an average of 18.0 eosinophils per HPF in the densest area, with a range of 0 to 68 and a median of 2. In the test tissues, there was an average of 25.2 eosinophils per HPF in the densest area, with a range of 0 to 38 and a median of 30.5 (*p* = 0.65, paired Student *t*-test). Additionally, the test samples showed increased scattered neutrophils and lymphocytes in the mucosa and submucosa, with one showing significant lymphocytic and neutrophilic inflammation (Figure 4C).

**Figure 5:**
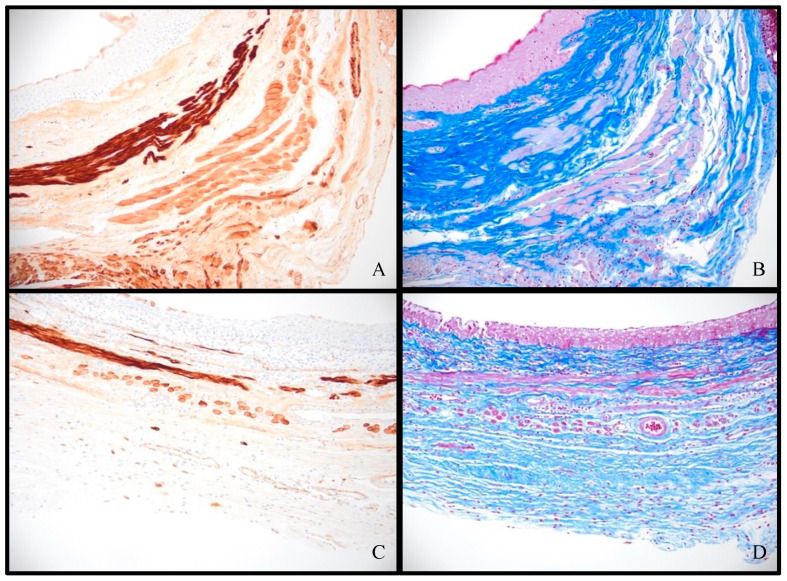
Desmin immunostain (left) and Masson’s trichrome (right) at 200× magnification demonstrating muscularis remodeling: (**A,B**) control sample 4; (**C,D**) test sample 6.

The trichrome stain highlighted the muscle (pink) and collagen (blue) layers in the vaginal wall (Figure 5A,C), while the desmin immunohistochemical stain used an anti-desmin antibody to highlight the desmin-protein-containing muscle in the vaginal wall (Figure 5B,D). While the muscle and collagen components are not stable in thickness throughout the length of the vaginal canal, a review of these stains shows an overall comparative thinning of the muscle layer of the vaginal wall in the test tissues, with an increase in fibrous connective tissue between the individual muscle fibers (Figure 5).

## 4. Discussion

Our study found that the VES device can induce tension-generated lengthening without any significant adverse tissue events. Through the VES, statistically significant vaginal lengthening was attained without statistically significant tissue thinning, which could signify some degree of new tissue formation over the course of the trial. Further investigation over a longer period of time is warranted to more accurately assess this possible ‘distraction vaginogenesis’. There is, however, important tissue remodeling occurring as a result of the stretching from the VES. Muscularis remodeling from tissue stretching was indicated by the Desmin immunostain and the Masson’s trichrome stains, which showed thinning of the muscularis with increased interspersed connective tissue between muscle fibers. The overall increased eosinophilia in the submucosal layer of the test tissues may indicate a specific, localized, subacute immune reaction to the foreign object VES device. Furthermore, the increased lymphocytic and neutrophilic infiltration in test sample 6 is notable because this is the rat that had the longest VES device. The degree of acute inflammation present in this sample may indicate that stretching was still ongoing at the time of sacrifice and tissue harvesting, while the subacute inflammation seen with the proportionally shorter VES devices may indicate that the rat vagina had physically adapted to the VES device. 

Future studies are warranted to more completely assess the histologic adaptation as well as to determine the VES length that would provide optimal amounts of tension on the vaginal canal tissue to induce non-destructive stretching. One VES device was cut to a longer length than the rest at 30 mm, resulting in significantly increased inflammation (test sample 6) compared to the other experimental tissues. The increased inflammation may have slowed any healing or adaptive processes from stretching, and we believe we may have attempted to stretch the tissue beyond an optimal capacity and consequently limited the amount of true expansion. This could explain why we achieved a lengthening of only 2 mm when there was a potential maximum increase of 7 mm. We will attempt to correct this in a future study through the use of serial implantations of our device at increasing lengths over a longer period of time until a desired vaginal canal length is reached, which will prevent overstretching and overwhelming the local tissue. Additionally, studies have shown that there is a normal variation in the degree of polymorphonuclear (PMN) leukocyte invasion depending on which stage of the estrous cycle a rat is experiencing [31]. There was no significant difference in the number of eosinophils per HPF in the test tissues versus control tissues, possibly reflecting the small sample size in this study. Future studies with larger sample sizes, as well as a determination of the stage of the rat estrous cycle and a baseline measurement of tissue PMN density for each stage, are needed to more accurately assess inflammatory changes associated with VES implantation. 

There are several limitations to our study, with the greatest being that we have used a rat model. However, the rat has been proven to be an excellent species to model both the gross anatomy and microanatomy of the human vagina and its supportive attachments [32,33]. Therefore, we anticipate that our findings may recapitulate what might be found in human clinical applications. Additionally, we are testing our device using rats with fully formed, normal-length vaginal canals. Our target patient population would have shorter or near-absent vaginal canals that may comprise tissue of varying composition, including less elastic scar tissue from prior attempts at treatment [11]. Moreover, our device requires some initial vaginal length in order to be placed, as a vaginal dimple alone may not provide enough space for the compressed VES device. However, we anticipate the VES to be used in those that do not have the motivation or favorable psychological state to completely and successfully achieve an adequate vaginal length through manual dilation alone. Once a space sufficient enough to fit the VES is gained, the spring-like VES device can replace the patient-input pressure for dilation to achieve the remaining length in those discouraged patients. Another limitation arose due to the rats’ ability to remove the device themselves. Four VES devices had been chewed out by the rats on day 6, compromising the intended length of the trial by one day. We will attempt to rectify this issue by re-wrapping their torsos with new tape at the first sign of loosening to further prevent their flexibility and isolate the area. Furthermore, we do not anticipate this type of difficulty in humans who would have chosen and consented to this method of treatment for themselves and would therefore be more compliant with maintaining the VES. Along with this idea of compliance, sutures may not have to be placed at all in human patients, as a rigid perineal guard worn within undergarments throughout the day and night may suffice to keep the VES from extruding during treatment. Nonetheless, future human studies are warranted to more fully assess the feasibility of prolonged insertion of the VES. 

We hope for the VES to be the bridge between manual dilation and surgery—a less invasive, more cost-effective means before total operative treatment with prolonged hospital stay is considered. We consider the insertion of our device to be non-surgical, as it does not require the patient to be under general anesthesia in the operating room. Insertion of the device in humans can theoretically be conducted as an outpatient procedural visit with sterile technique under local anesthesia. Furthermore, follow up on its location and progress can be performed outpatient in routine visits.

Our VES device has the potential to offer a more emotionally comfortable treatment option for vaginal atresia and the construction of a neovagina [5]. Existing treatment methods often carry the emotional burden of reminding individuals of their differences. By utilizing the VES, we aim to relieve this sense of ‘abnormality’ by reducing the frequency of reminders associated with the daily self-application of therapy. The VES device is designed to generate the required pressure for canal stretching automatically, effectively replacing the manual effort typically required from the patient. This innovation aims to simplify the process, minimize needs for procedural compliance, and reduce negative emotional consequences associated with the treatment process.

## 5. Conclusions

The VES is a unique approach for elongating the vaginal or neovaginal canal in individuals with vaginal atresia through the process of ‘distraction vaginogenesis’. Additionally, our device was able to achieve vaginal stretching without any significant adverse tissue events.

Something to consider in our future studies is the actual material of our VES. One study found success in maintaining a vaginal space without a skin graft after a modified Abbe–McIndoe procedure using a new poly-lactic acid (PLA) prosthesis [34]. PLA is a biodegradable, biocompatible polymer commonly used in regenerative medicine and tissue engineering, making it an effective scaffold or mold material [34]. However, we need our device to have some flexibility and elasticity to create the spring-like design needed for force generation. We may therefore consider blending PLA with thermoplastic polyurethane (TPU), another biocompatible polymer commonly used for its flexible properties [35]. In using a PLA-TPU blend, we may 3-D print a VES of our own design that has a semi-rigid structure with elastic properties suitable for our study goals [35].

One question left unanswered in this trial is whether or not the new vaginal length is maintained over a longer period of time or if the vaginal canal contracts down to its original length. Moving forward, further in vivo studies are required to adequately assess this ‘rubber band’ effect. These next steps will also allow us to assess the optimal time interval for VES insertion as well as whether serial placement of longer VES devices would be more efficient for those requiring greater added length. Moreover, we plan to impregnate the VES device with estrogen, vascular endothelial growth factor (VEGF), or GLP-2 to aid mucosal stimulation as well as to enhance the neovaginal tissue architecture during the trials. For now, further studies are required to determine the length and number of time intervals in order to most fully optimize vaginal expansion through the process of ‘distraction vaginogenesis’.

## 6. Patents

The authors (J.S.A., D.S., and G.S.) have disclosed this technology (“Tissue Expander Sleeve and Treatments for Short Bowel Syndrome, Vaginal Stenosis, Vaginal Agenesis/Atresia and Neovaginal Dilation”) to the LSU Health Office of Sponsored Programs and Technology Transfer.

## Figures and Tables

**Table 1:** Vaginal canal lengths (VLs) before VES insertion and after VES removal.

	1	2	3	4	5	6
VES Length (mm)	25	27	27	27	25	30
Pre-insertion VL (mm)	18	23	18	18	20	23
Post-insertion VL (mm)	25	24	22	24	23	25
Expansion (%)	38.9	4.3	22.2	33.3	15.0	8.7

## Data Availability

The data presented in this study are available on request from the corresponding author.

## References

[1] Nakhal R.S., Creighton S.M. (2012). Management of vaginal agenesis. J. Pediatr. Adolesc. Gynecol..

[2] Oelschlager A.M., Debiec K., Appelbaum H. (2016). Primary vaginal dilation for vaginal agenesis: Strategies to anticipate challenges and optimize outcomes. Curr. Opin. Obstet. Gynecol..

[3] Committee on Adolescent Health Care (2018). ACOG Committee Opinion No. 728 Summary: Müllerian Agenesis: Diagnosis, Management, and Treatment. Obstet. Gynecol..

[4] Acién P., Acién M. (2007). Malformations of the female genital tract and embryological bases. Curr. Women’s Health Rev..

[5] Edmonds D.K., Rose G.L., Lipton M.G., Quek J. (2012). Mayer-Rokitansky-Küster-Hauser syndrome: A review of 245 consecutive cases managed by a multidisciplinary approach with vaginal dilators. Fertil. Steril..

[6] Herlin M.K., Petersen M.B., Brännström M. (2020). Mayer-Rokitansky-Küster-Hauser (MRKH) syndrome: A comprehensive update. Orphanet J. Rare Dis..

[7] Fulare S., Deshmukh S., Gupta J. (2020). Androgen Insensitivity Syndrome: A rare genetic disorder. Int. J. Surg. Case Rep..

[8] Hughes I.A., Davies J.D., Bunch T.I., Pasterski V., Mastroyannopoulou K., MacDougall J. (2012). Androgen insensitivity syndrome. Lancet.

[9] Kang J., Chen N., Song S., Zhang Y., Ma C., Ma Y., Zhu L. (2020). Sexual function and quality of life after the creation of a neovagina in women with Mayer-Rokitansky-Küster-Hauser syndrome: Comparison of vaginal dilation and surgical procedures. Fertil. Steril..

[10] Callens N., De Cuypere G., De Sutter P., Monstrey S., Weyers S., Hoebeke P., Cools M. (2014). An update on surgical and non-surgical treatments for vaginal hypoplasia. Hum. Reprod. Update.

[11] Ozkan O., Erman Akar M., Ozkan O., Doğan N.U. (2011). Reconstruction of vaginal agenesis. Ann. Plast. Surg..

[12] Abbe R. (1898). New method of creating a vagina in a case of congenital absence. Med. Rec..

[13] McIndoe A. (1950). The treatment of congenital absence and obliterative condition of the vagina. Br. J. Plast. Surg..

[14] Davydov S.N. (1969). Colpopoiesis from the peritoneum of the uterorectal space. Obstet. Gynecol..

[15] Davydov S.N., Zhvitiashvili O.D. (1974). Formation of vagina from peritoneum of Douglas pouch. Acta Chir. Plast..

[16] Vecchietti G. (1965). Neovagina nella sindrome di Rokitansky-Kuster-Hauser. Attual. Ostet. Ginecol..

[17] Vecchietti G. (1979). The neovagina in the Robitansky-Kuster-Hauser syndrome. Rev. Medicale Suisse Rom..

[18] Bach F., Glanville J.M., Balen A.H. (2011). An observational study of women with müllerian agenesis and their need for vaginal dilator therapy. Fertil. Steril..

[19] Frank R. (1938). The formation of an artificial vagina without operation. Am. J. Obstet. Gynecol..

[20] Ismail-Pratt I.S., Bikoo M., Liao L.M., Conway G.S., Creighton S.M. (2007). Normalization of the vagina by dilator treatment alone in complete androgen insensitivity syndrome and Mayer-Rokitansky-Kuster-Hauser syndrome. Hum. Reprod..

[21] Passos I.D.M.P.E., Britto R.L. (2020). Diagnosis and treatment of müllerian malformations. Taiwan. J. Obstet. Gynecol..

[22] Gargollo P.C., Cannon G.M., Diamond D.A., Thomas P., Burke V., Laufer M.R. (2009). Should progressive perineal dilation be considered first line therapy for vaginal agenesis?. J. Urol..

[23] Roberts C.P., Haber M.J., Rock J.A. (2001). Vaginal creation for müllerian agenesis. Am. J. Obstet. Gynecol..

[24] Nadarajah S., Quek J., Rose G.L., Edmonds D.K. (2005). Sexual function in women treated with dilators for vaginal agenesis. J. Pediatr. Adolesc. Gynecol..

[25] Rock J.A., Reeves L.A., Retto H., Baramki T.A., Zacur H.A., Jones H.W. (1983). Success following vaginal creation for Müllerian agenesis. Fertil. Steril..

[26] Meyer H., Trosclair L., Clayton S.D., O’Quin C., Connelly Z., Rieger R., Sorrells D. (2023). ‘Distraction Vaginogenesis’: Preliminary Results Using a Novel Method for Vaginal Canal Expansion in Rats. Bioengineering.

[27] Clayton S., Alexander J.S., Solitro G., White L., Villalba S., Winder E., Boudreaux M., Veerareddy P., Dong E., Minagar A. (2022). Self-expanding intestinal expansion sleeves (IES) for short gut syndrome. Pediatr. Surg. Int..

[28] Koga H., Sun X., Yang H., Nose K., Somara S., Bitar K.N., Owyang C., Okawada M., Teitelbaum D.H. (2012). Distraction-induced intestinal enterogenesis: Preservation of intestinal function and lengthening after reimplantation into normal jejunum. Ann. Surg..

[29] Sengupta P. (2013). The Laboratory Rat: Relating Its Age With Human’s. Int. J. Prev. Med..

[30] Moolenbeek C., Ruitenberg E.J. (1981). The ‘Swiss roll’: A simple technique for histological studies of the rodent intestine. Lab. Anim..

[31] Daly T.J.M., Kramer B. (1998). Alterations in rat vaginal histology by exogenous gonadotrophins. J. Anat..

[32] McGuire J.A., Monclova J.L., Coariti A.C.S., Stine C.A., Toussaint K.C., Munson J.M., De Vita R. (2021). Tear propagation in vaginal tissue under inflation. Acta Biomater..

[33] Moalli P.A., Howden N.S., Lowder J.L., Navarro J., Debes K.M., Abramowitch S.D., Woo S.L. (2005). A rat model to study the structural properties of the vagina and its supportive tissues. Am. J. Obstet. Gynecol..

[34] Acién P., Nohales-Alfonso F.J., Sánchez-Ferrer M.L., Sánchez-Lozano M., Navarro-Lillo V., Acién M. (2019). Clinical pilot study to evaluate the neovaginal PACIENA prosthesis^®^ for vaginoplasty without skin grafts in women with vaginal agenesis. BMC Women’s Health.

[35] Samat A., Abdul Hamid Z.A., Jaafar M., Ong C.C., Yahaya B.H. (2023). Investigation of the in vitro and in vivo biocompatibility of a Three-Dimensional printed thermoplastic Polyurethane/Polylactic Acid blend for the development of tracheal scaffolds. Bioengineering.

